# Pharmacological Effects of *Gami-Yukmijihwang-Tang* on the Lipopolysaccharide-Induced Hippocampus Oxidation and Inflammation via Regulation of Sirt6

**DOI:** 10.3390/ph15030293

**Published:** 2022-02-28

**Authors:** Jie-Yoon Kang, Jong-Suk Lee, In-Chan Seol, Yoon-Sik Kim, Miso S. Park, Ho-Ryong Yoo

**Affiliations:** 1 Department of Cardiology and Neurology of Korean Medicine, College of Korean Medicine, Daejeon University, Daejeon 34520, Korea; cardoestrellado1@gmail.com (J.-Y.K.); seolinch@dju.kr (I.-C.S.); yoonsik@dju.kr (Y.-S.K.); 2 Biocenter, Gyeonggido Business & Science Accelerator (GBSA), Suwon 16229, Korea; leejs@gbsa.or.kr

**Keywords:** *Yukmijihwang-Tang*, neuroinflammation, oxidative stress, hippocampus, sirtuin6, nuclear factor erythroid 2–related factor 2

## Abstract

*Yukmijihwang-Tang* is widely used in traditional Korean medicine to treat age-related disorders. In the present study, we re-prescribed *Gami-Yukmijihwang-Tang* (YJT), which is slightly modified from *Yukmijihwang-Tang* by adding more medicinal plants to evaluate its pharmacological effects on underlying mechanisms against repeated lipopolysaccharide (LPS)-injection-induced neuroinflammation in the hippocampus regions. C57BL/6J male mice (16–24 weeks old) were divided into six groups: (1) the control group (DW with 0.9% saline injection), (2) LPS group (DW with LPS injection), YJT groups ((3) 100, (4) 200, or (5) 400 mg/kg of YJT with LPS injection), and (6) glutathione (GSH) group (100 mg/kg of GSH with LPS injection), respectively. Mice were orally administrated with various doses of YJT or glutathione (GSH) for the first five days. Neuroinflammation in the hippocampus region was induced by repeated injection of LPS during the last three days. As predicted, LPS not only increased oxidative stress–related markers including malondialdehyde, 4-hydroxynonenal, nitrotryptophan, and hydrogen peroxide, but also drastically enhanced inflammatory reactions including nitric oxide, inducible nitric oxide synthase, p65, and toll-like receptor 4, respectively. YJT administration, on the other hand, notably decreased the above pathological alterations by enhancement of antioxidant capacities such as superoxide dismutase and catalase activities. To explain the underlying pharmacological actions of YJT, we focused on a representative epigenetic regulator, a nicotinamide adenine dinucleotide + (NAD+)–dependent chromatin enzyme, Sirtuin 6 (Sirt6). Neuroinflammation in hippocampus regions depleted Sirt6 at the protein level and this alteration directly affected the nuclear factor erythroid 2–related factor (Nrf2)/hemeoxygenase (HO)-1 signaling pathway in the LPS group; however, YJT significantly recovered the Sirt6 protein levels, and it could recover the abnormal status of Nrf2/HO-1 signaling pathways in the hippocampus regions. Additionally, Sirt6 led to the up-regulation of GSH sub-enzymes of mRNA expression and protein levels of total GSH content. These findings suggest that YJT can protect against LPS-induced neuroinflammation and oxidative stress by regulating the Sirt6-related pathways and normalizing the GSH redox cycle.

## 1. Introduction

The global burden of Alzheimer’s disease (AD) and other dementias have been continuously increasing. Globally, the number of people living with dementia more than doubled between 1990 and 2016. By 2050, the number of dementia patients is expected to reach 100 million. AD and dementia affect not only individuals but also their families and healthcare systems. Therefore, it is urgent to find preventive and curative dementia treatments [1].

Traditionally, AD and other neurodegenerative diseases have been regarded as a disease of the brain tissue. However, it has been recently discovered that immune-senescence and neuroinflammation play critical roles in the pathogenesis of neurodegenerative diseases [2]. Inflamm-aging is a common phenomenon in the elderly population, which is characterized by the continuously stimulated immune system that leads to chronic subclinical inflammation. Serum levels of pro-inflammatory cytokines such as tumor necrosis factor-α (TNF-α), interleukin-1β (IL-1β), and interleukin-6 (IL-6) are generally elevated in the elderly [3]. Because such pro-inflammatory cytokines can damage and affect the permeability of the blood–brain barrier, as well as cross it, systemic inflammation can directly promote neuroinflammation [4].

Neuroinflammation is one of the primary causes of synaptic loss in the brain, which can lead to cognitive impairment. Both acute and chronic neuroinflammation are major contributors to a wide range of neurodegenerative diseases, including AD, Parkinson’s disease, and Huntington’s disease [5,6], as well as multiple sclerosis and amyotrophic lateral sclerosis [7,8,9]. Particularly, the hippocampus is the main target of pathogenesis AD among various regions of brain tissue [10,11] because this area plays a critical function for neuronal regeneration, also known as neurogenesis [12]. Numerous pathophysiological factors play a role in the pathological progression of neuroinflammation in complex ways in brain tissue, particularly the hippocampus regions [6,13]. During neuroinflammation, microglia cells are activated and infiltrated through the hippocampus regions, then they abnormally release pro-inflammatory cytokines including TNF-α, Il-1β, and Il-6, as well as oxidative stress mediators such as reactive oxygen species (ROS) and reactive nitrogen species (RNS), respectively [14,15,16,17,18,19]. The molecules listed above can be harmful to neurons because they can directly and extensively react with normal neurons, leading to their damage and death. However, the exact mechanisms of neuroinflammation remain unclear. Therefore, it is critical to elucidate the precise pathological mechanisms and develop drug therapeutics that can prevent and treat neuroinflammation-related disorders.

Meanwhile, there are several candidate herbal species and prescriptions in traditional Korean medicine for inflammatory diseases. Among these, *Yukmijihwang-Tang* has displayed a neuroprotective effect in various animal models of neuronal disorders by enhancing antioxidant capacity, reducing neuroinflammation, and improving memory ability [20,21,22]. In this study, we developed a slightly modified version of the *Yukmijihwang-Tang* and intended to enhance its anti-neuroinflammatory properties, called *Gami-Yukmijihwang-Tang* (YJT), against a mouse model of brain injury, caused by intraperitoneal (i.p.) injection of the lipopolysaccharide (LPS), a gram-negative bacterial endotoxin. We discovered that this model was suitable for inducing neuroinflammation [23,24,25], which is particularly related to the similar pathophysiological state of AD [26,27], and YJT significantly reduced microglial activation-mediated neuronal cell damage in hippocampal areas, particularly by regulating Sirtuin 6 (Sirt6) protein levels in the hippocampus.

## 2. Results

### 2.1. YJT Ameliorates LPS-Induced Oxidation in the Hippocampus Region

**Figure 1:**
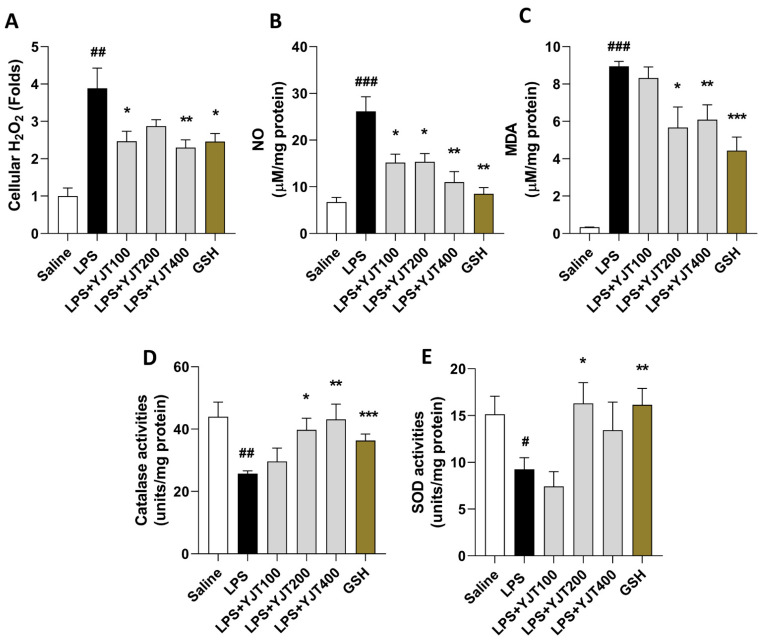
YJT ameliorated LPS–induced hippocampus oxidation in the protein levels. Mice were sacrificed and brain tissues were removed after the final LPS injection. The hippocampus was isolated from brain tissue, and protein lysate was prepared for further analysis (*n* = 6–9 in each group). Hippocampus cellular levels of (**A**) H_2_O_2_, (**B**) NO, (**C**) MDA, (**D**) catalase activities, and (**E**), SOD activities were measured. The data were expressed as mean ± SEM. # *p* < 0.05, ## *p* < 0.01 and ### *p* < 0.001 for Con vs. LPS, * *p* < 0.05, ** *p* < 0.01, and *** *p* < 0.001 for LPS vs. drug–administrated groups.

We observed LPS-induced brain injury of hippocampus regions by abnormal increases of oxidative stress in protein levels including H_2_O_2_, NO, and MDA in the LPS group compared to the control group (*p* < 0.01 or 0.001). Administration with YJT, the above oxidative stress–related molecules and final products were significantly decreased as compared to the LPS group (*p* < 0.05 or 0.01 in Figure 1A–C) by enhancement of antioxidant component activities such as catalase and SOD, respectively (*p* < 0.05 or 0.01 in Figure 1D,E).

**Figure 2:**
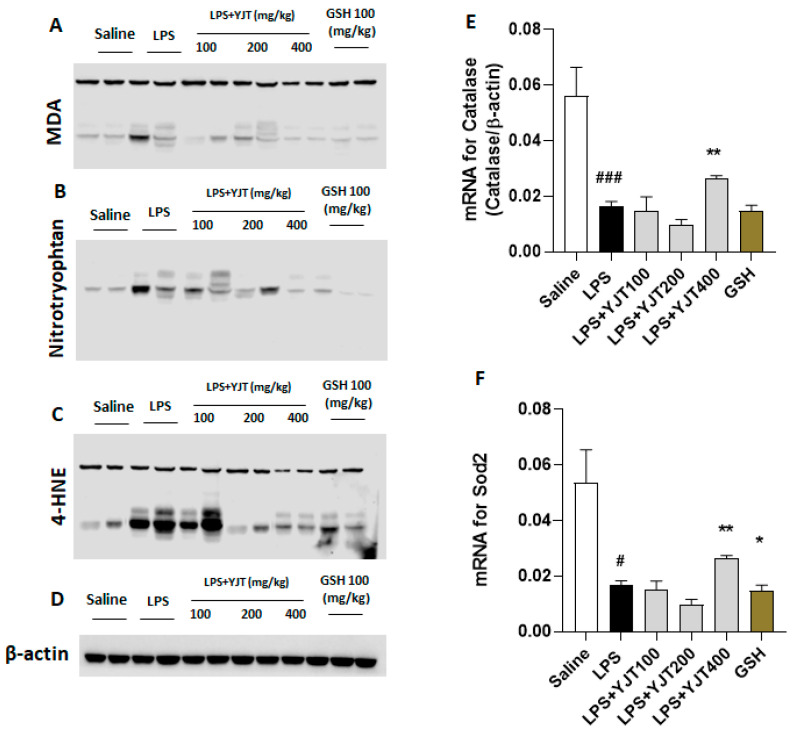
Western blot analysis of oxidation–related proteins and mRNA expression levels of antioxidant enzymes in hippocampus regions. Mice were sacrificed and brain tissues were removed after the final LPS injection. The hippocampus was isolated from brain tissue and prepared either protein lysates (*n* = 6–9 to each group) or mRNA (*n* = 4–6 to each group) samples were prepared for further analysis. Oxidative stress–related proteins including (**A**) MDA, (**B**) nitrotryptophan, and (**C**) 4–HNE were performed by Western blot analysis (*n* = 4 to each group). (**D**) β–actin was used as internal control. mRNA expression levels of antioxidant enzymes such as (**E**) catalase and (**F**) Sod2 were measured (*n* = 4–6 to each group). The data were expressed as mean ± SEM. # *p* < 0.05, and ### *p* < 0.001 for Con vs. LPS, * *p* < 0.05, and ** *p* < 0.01 for LPS vs. drug–administrated groups.

Western blot analyses of hippocampus protein levels showed that YJT effectively worked against LPS-overload mediated oxidation by decreases of MDA, 4-HNE, and nitrotryptophan levels as compared with the LPS group (Figure 2A–D). Additionally, mRNA expression levels of antioxidant enzymes such as catalase and Sod2 also supports these antioxidant properties of YJT by up-regulation of gene expression levels as compared to the LPS group (*p* < 0.01 in Figure 2E,F).

### 2.2. YJT Attenuates Inflamed Cells Infiltrations in the Hippocampus Region

**Figure 3:**
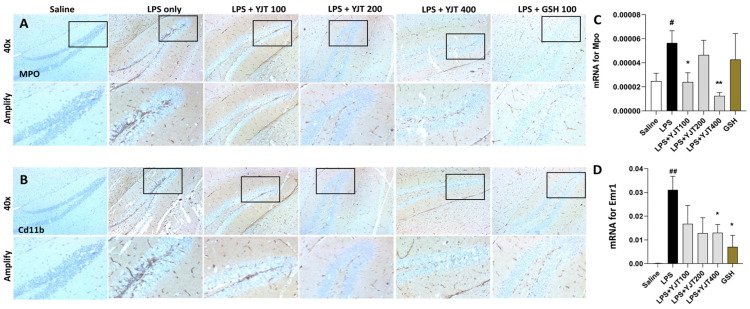
Immunohistochemistry analysis and qPCR analysis for neuroinflammatory factors. After the last injection of LPS, mice were sacrificed and brain tissues were removed and stored in the neutral formalin solutions for further analysis. Immunohistochemistry analysis was performed specifically against (**A**) MPO and (**B**) Cd11b (*n* = 3–4 to each group), and qPCR analysis for Mpo (**C**) and Emr1 (**D**) (*n* = 4–6 to each group). The data were expressed as mean ± SEM. # *p* < 0.05, and ## *p* < 0.01 for Con vs. LPS, * *p* < 0.05, and ** *p* < 0.01 for LPS vs. drug–administrated groups. The scale bars on the 40× indicate 250 µm and amply pictures for 100 µm, respectively.

Next, we confirmed anti-neuroinflammatory effects of YJT by completion of IHC analysis against MPO and Cd11b, which are known as microglial cell markers, respectively. As predicted, LPS injection markedly increased both MPO and C11b positive signals through hippocampus areas, especially regions of *dentate gyrus* (DG); however, YJT remarkably decreased these alterations (Figure 3A,B). Hippocampus gene expression levels of both Mpo and Emr1 are coincided with the IHC analysis results (Figure 3C,D).

### 2.3. YJT Inhibit Neuroinflammation via Inactivation of Microglia Cell Activation

**Figure 4:**
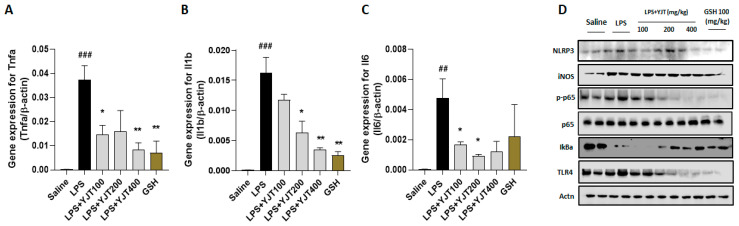
Western blot and gene expression analysis of LPS–induced hippocampal injury, resulting inflammatory signaling pathways. Mice were sacrificed and brain tissues were removed after the final LPS injection. The hippocampus was isolated from brain tissue and prepared either protein lysates (*n* = 6–9 to each group) or mRNA (*n* = 4–6 to each group) samples were prepared for further analysis. qPCR analysis was used to determine the mRNA expression levels of pro–inflammatory cytokines including (**A**) Tnfa, (**B**) Il1b, and (**C**) Il6. Western blot analysis of (**D**) NLRP3, iNOS, p–p65, p–65, IκBα, TLR4, and actinin were performed (*n* = 4 to each group). The data were expressed as mean ± SEM. ## *p* < 0.01 and ### *p* < 0.001 for Con vs. LPS, * *p* < 0.05, and ** *p* < 0.01 for LPS vs. drug–administrated groups.

We further confirmed that mRNA expression levels of pro-inflammatory cytokines in TNF-α, IL-1β, and IL-6 in hippocampus regions were significantly up-regulated by LPS injection as compared to the control group (*p* < 0.01 or 0.001), whereas these abnormal alterations were markedly normalized by YJT treated group (*p* < 0.05 or 0.01 for Figure 4A–C).

NLRP3 inflammasome is involved in LPS-challenged hippocampus regions, which was evidenced by Western blot analysis, and other inflammation associated proteins including iNOS, p65 (NF-κB), IκBα, and TLR4 support the microglial cells activation (Figure 4D). Administrations with YJT, however, remarkably normalized the above-listed abnormalities in the hippocampus protein levels (Figure 4D).

### 2.4. YJT Normalized GSH Redox Cycle by Regulation of Sirt6/Nrf2/HO-1 Signaling Pathway

**Figure 5:**
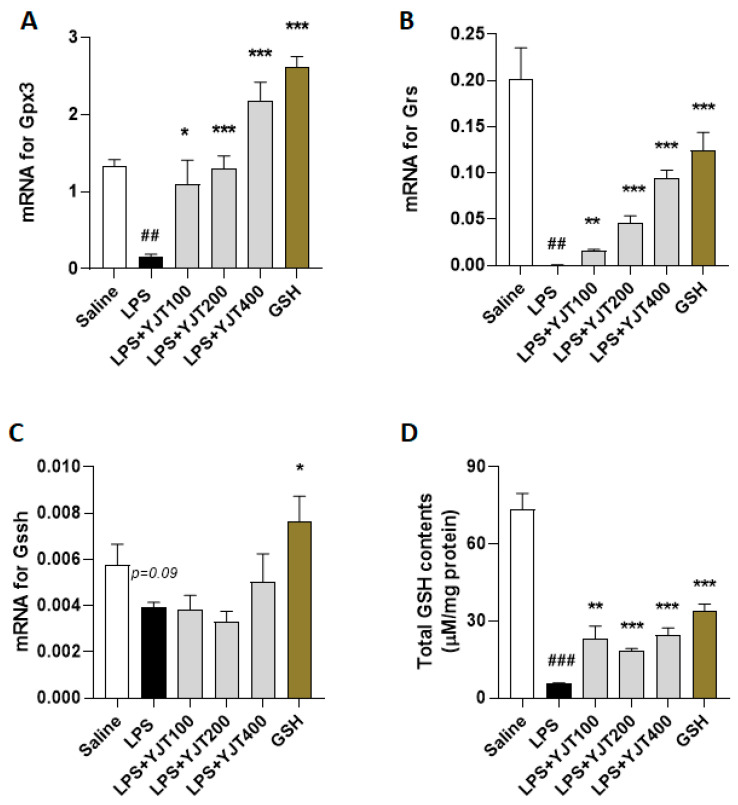
Effects of YJT on glutathione redox cycles in the hippocampus. Mice were sacrificed and brain tissues were removed after the final LPS injection. The hippocampus was isolated from brain tissue and prepared protein lysates (*n* = 6–9 to each group) or mRNA (*n* = 4–6 to each group) samples. Gene expression analysis of (**A**) Gpx3 (**B**) Gsr, (**C**) Gssh, and (**D**) protein levels of total GSH contents were measured. The data were expressed as mean ± SEM. ## *p* < 0.01 and ### *p* < 0.001 for Con vs. LPS, * *p* < 0.05, ** *p* < 0.01, and *** *p* < 0.001 for LPS vs. drug–administrated groups.

Next, we addressed the possible pharmacological actions of YJT and observed that gene expression levels of glutathione peroxidae-3 (Gpx3), glutathione reductase (Gsr), and glutathione synthase (Gssh) in the LPS group were significantly down-regulated as compared to the control group (*p* < 0.01 for Figure 5A–C), whereas administration with YJT significantly normalized these abnormalities of gene expression levels (*p* < 0.05, *p* < 0.01, or *p* < 0.01 for Figure 5A–C). Total GSH contents in the hippocampus regions are also correlated to the gene expression results (*p* < 0.01 or 0.001 for Figure 5D).

**Figure 6:**
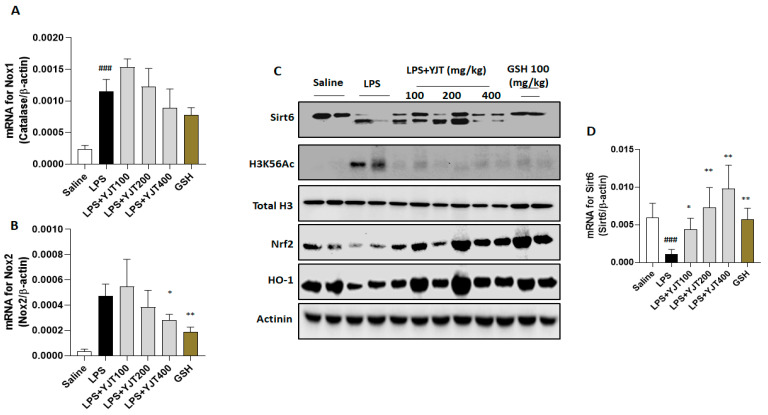
Pharmacological actions of YJT via mediation of Sirt6/Nrf2/HO–1 signaling pathways. Mice were sacrificed and brain tissues were removed after the final LPS injection. The hippocampus was isolated from brain tissue and prepared either protein lysates (*n* = 6–9 to each group) or mRNA (*n* = 4–6 to each group) samples were prepared for further analysis. qPCR analysis was used to determine the mRNA expression levels of (**A**) Nox1 and (**B**) Nox2. Western blot analysis of (**C**) Sirt6, H3K56Ac, Total H3, Nrf2, HO–1, and Actinin were performed (*n* = 4 to each group). Finally, the gene expression level of (**D**) Sirt6 was quantified with qPCR. The data were expressed as mean ± SEM. ### *p* < 0.001 for Con vs. LPS, * *p* < 0.05, and ** *p* < 0.01 for LPS vs. drug–administrated groups.

Meanwhile, when compared to the control group, LPS injection significantly increased nicotinamide adenine dinucleotide phosphate (NADPH) oxidase (Nox) 1 and Nox2 mRNA expression in the hippocampus (*p* < 0.001). YJT administration at a dose of 400 mg/kg did not significantly normalize Nox2 overexpression (*p* < 0.05 for Figure 6A), but it significantly down-regulated the above abnormality of Nox2 (*p* < 0.05 for Figure 6B).

LPS injection significantly reduced both protein and mRNA expression levels of Sirt6 in the hippocampus as compared to the control group (*p* < 0.05 for Figure 6C,D); however, YJT administration significantly increased Sirt6 mRNA expression in a dose-dependent manner as compared to the control group (*p* < 0.05 for Figure 6D). The Western blot analysis provides additional support to this finding, as Sirt6 protein levels were reduced after LPS injection, but this was prevented in drug-treated groups (Figure 6C). The protein levels of histone H3 Lysine 56 acetylation (H3K56Ac) were increased, but YJT administration decreased this alteration as compared with the LPS group (Figure 6C). To comprise of the above properties, nuclear factor erythroid 2–related factor 2 (Nrf2) was significantly depleted in the LPS group, compared to the control group. Along with Nrf2, HO-1 was significantly depleted as well after LPS injection, whereas YJT administration significantly prevented Nrf2 and HO-1 depletion at the protein levels of hippocampus regions (Figure 6C).

## 3. Discussion

Microglial activation and the release of ROS, RNS, and pro-inflammatory cytokines during neuroinflammation are largely responsible for neurodegenerative diseases [28,29,30], and these inflammatory responses are deeply linked to neuronal oxidative stress [31,32]. An immunosenescence, a progression of immune dysfunctions according to the aging, is featured by remarkable decreases of cell-mediated immunity with continuous inflammatory reactions called ‘inflamm-aging’ [33]. Although an accurate mechanism for the immunosenescence between neurodegeneration and inflammation remains incompletely, neuroinflammation is a critical factor to control a wide range of age-related neurodegenerative diseases [4].

Through the current study, we employed a murine model of LPS-induced hippocampal injuries to model systemic inflammation-induced neuroinflammation. LPS is well known for activation of the macrophage, a type of innate immune cell with phagocytic function against harmful xenobiotics that can provoke inflammation and oxidative stress. The vicious cycle of inflammation can be rapidly accelerated by activation of cell death signals. Therefore, reducing inflammatory response and preventing oxidative stress are critical components of therapeutic strategies for various inflammatory diseases [16,24,25,34,35]. Besides, LPS can promote systemic inflammation by stimulations of microglia activation. Peripheral injection of LPS in rodent models caused a disruption of the blood brain barrier, as well as a significant increase in brain TNF-α, IL-1β, and IL-6 levels [36]. In a previous study, systemic LPS stimulation in mice resulted in morphologically activated microglia in the hippocampus and other wide brain regions after 48 h [37].

Our goal, in the present study, was to identify potential pathological mechanisms of LPS-induced neurotoxicity, especially in the aspect of neuroinflammation and oxidative damage, which are main pathological features of neurovegetative diseases, particularly AD [12,13]. Furthermore, we also proposed to reveal the pharmacological properties of YJT with underlying mechanisms. YJT mainly exhibit its effects on the decreases of microglial activation, which was evidenced by abnormal increases in hydrogen peroxide, nitric oxide, and MDA levels in the hippocampus (Figure 1A–C). Additionally, Western blot analysis for MDA, 4-HNE, and nitrotryptophan supports the above effects (Figure 2A–D). These pharmacological properties mainly would be attributed by increasing the endogenous antioxidant components such as SOD and catalase activities in both hippocampus protein and gene expression levels (Figure 2D,E). We further revealed effects of YJT by reduction of neuroinflammation, which were proved by decreases of positive signals for microglial activation markers (Figure 3B and Figure 4D) and their related gene expression levels (Figure 3C,D and Figure 4A–C).

According to the proof for the pharmacological effects of YJT, especially increases of antioxidant capacities, we further measured promising endogenous antioxidant component, GSH and its related sub-enzymatic cycles. As the most essential antioxidant component, GSH is a major peptide in brain tissue for cellular detoxification against oxidation. A compromised GSH redox cycle in brain tissue is closely linked to oxidative stress, which is related to a variety of neurological diseases [38,39]. The mRNA expression levels of Gpx3, Gsr, and Gssh were significantly lower in the LPS group compared to the control group, according to evidence from qPCR. Meanwhile, YJT significantly up-regulated these animalities (Figure 5A–C). YJT treatment clearly improved the hippocampal protein levels of total GSH contents, which had been deteriorated due to LPS injection (Figure 5D).

Meanwhile, the Nox is found in cell membranes and is known to catalyze the formation of superoxide free radicals, which can aggravate oxidative stress, exacerbate inflammation, and cause neuronal injury and death. Therefore, Nox inhibition is known to be neuroprotective [40]. In a previous study, exposure to LPS increased Nox2 expression and induced oxidative stress in the hippocampal neurons [41]. In this study, by performance of qPCR analysis, we revealed both Nox1 and Nox2 mRNA expression levels were significantly up-regulated by LPS injection; whereas, at a dose of 400 mg/kg, YJT significantly normalized such Nox2 over-expression in the hippocampus gene levels (Figure 6B). At a dose of 400 mg/kg, YJT also showed the tendency of down-regulation of Nox1 in the gene expression levels, but the difference was not statistically significant (Figure 6A). These interesting results might be selective properties of YJT in hippocampus neuroinflammatory condition.

Previous studies have reported that early brain injury can be detected primarily by heightened oxidative stress signals, especially abnormally elevated ROS levels during pathological conditions, and this status is intimately linked to antioxidant signaling pathways, particularly Nrf2/HO-1 axis, which is also involved in the regulation of numerous biological processes, including oxidation and inflammation [32,42,43]. The Western blot analysis showed that LPS-injection-induced neurotoxicity was mainly caused by alterations of Nrf2/HO-1 protein levels in the hippocampus, and YJT significantly restored them (Figure 6C). These redox status regulatory enzymes are also closely linked to epigenetic regulators, such as the Sirtuin family members [44,45,46,47]. Therefore, we focused on epigenetic regulators, particularly Sirt6 in the hippocampus. The LPS group’s mRNA expression levels displayed a strong down-regulation of Sirt6, and Western blot analysis confirmed the qPCR results (Figure 6C,D).

Sirt6 is a deacetylase that functions as an epigenetic regulator of genes involved in inflammation, DNA damage, and aging [44,45,46,47]. Recent studies have evidenced that Sirt6 plays a role in a variety of diseases through regulating anti-inflammatory molecules and antioxidant proteins [48]. Nrf2/HO-1 signaling pathway is deeply related to regulation of GSH system and it is mainly targeted by Sirt6 [49,50]. The pathophysiological roles of Sirt6 in the brain tissue, specifically neuroinflammation and AD, is now highlighted in neuronal degenerative disease with inflammatory status [51]. Interestingly, administration with YJT remarkably normalized the alterations in Sirt6 with histone H3 acetylation modifications (Figure 6E,F). The neurotoxicity caused by LPS injection could be ameliorated by YJT administration. Our finding is also supported by previous studies, which reveal the roles of Sirt6 on neuroinflammation via amelioration of microglial activation as well as preventing chromatin alterations in ischemic brain injuries [52,53]

Our previous studies reported pharmacological effects of *Yukmijihwang-Tang* against chronic restrain stress–induced hippocampal excitotoxicity by enhancement of phosphor-cAMP response element-binding protein (p-CAMP) and brain-derived neurotrophic factor (BDNF), which were regulated by NRF2/HO-1 signaling pathway [21], and we also observed that scopolamine-induced memory-deficit mice improved their cognitive activities by administration with *Yukmijihwang-Tang* by normalization of acetylcholine esterase activities in hippocampus protein levels as well as neuronal regenerations [22]; however, there is no accurate clue which molecule would be implicated to modulate the above conditions. Thus, we re-prescribed *Yukmijihwang-Tang* by adding more medicinal plants to anticipate its obvious pharmacological effects with clear mechanism using neuroinflammation condition by focusing on the epigenetic regulator, especially Sirt6, which is a key modulator of inflammatory condition of hippocampus regions [54,55].

## 4. Materials and Methods

### 4.1. Preparation and Fingerprinting Analysis of YJT

**Figure 7:**
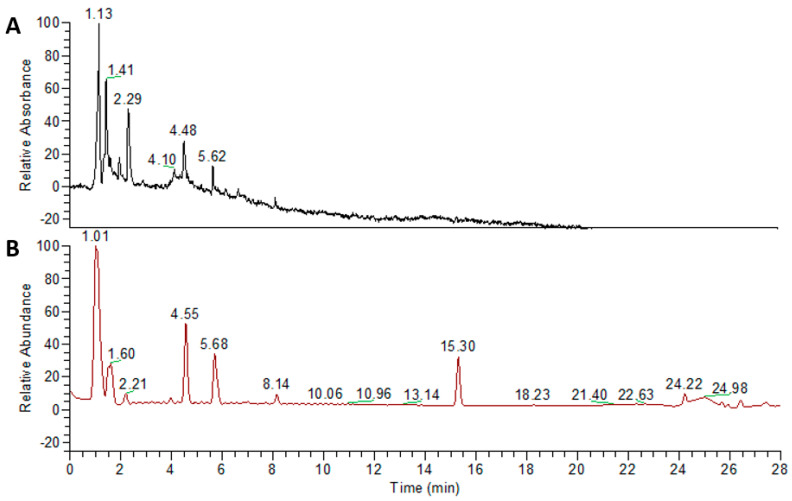
Fingerprinting analysis of YJT using ultra–high–performance liquid chromatography (UHPLC). To elucidate the chemical compositions of YJT, fingerprinting analysis was performed using Accela ultra–high–performance liquid chromatography (UPLC–MS/MS) with two different modes: (**A**) PDA and (**B**) MS+ modes, respectively.

We slightly modified the original prescription of YJT in this study by adding and changing some herbal components. The herbal components of our YJT are listed in Supplementary Table S1. A total of 86 g of the herbal mixture was decocted with distilled water (DW) at 85 °C for 6 h, passed through a 300-mesh filter, precipitated for 1.5 h, cooled at room temperature (RT), and then stored at −80 °C overnight. The lyophilization process was carried out for 72 h and the powder was collected with a final yield of 10.65% (*w*/*w*). YJT fingerprinting was performed using a high-resolution LTQ-Orbitrap XL mass spectrometer in conjunction with an Accela ultra-high-performance liquid chromatography (UHPLC) system (Thermo Fisher Scientific, Waltham, MA, USA). We used the ACQUITY UPLC BEH C18 column (150 × 2.1 mm, 1.7 μm; Waters Corporation, Milford, MA, USA) to separate biomolecules based on their polarity. The flow rate was 0.4 mL/min, and the injection volume was 3 µL. The solvent gradient conditions of binary mobile phases with solvent A (DW with 0.1% (*v*/*v*) formic acid) and solvent B (acetonitrile with 0.1% (*v*/*v*) formic acid) were as follows: 95% A/5% B at 0–1 min, 30% A/70% B at 20 min, 0% A/100% B at 24 min, 95% A/5% B at 27.1 min, and 95% A/5% B at 30 min. Components were detected from 200 to 600 nm. The MS conditions in positive and negative HESI mode were optimized at the following values: the capillary temperature at 300 °C, the capillary voltage at 35 V, and the spray voltage at 4.0 kV. Data were acquired and processed with Xcalibur software (Figure 7). Metabolite profile data based on reference literature, high-resolution mass, and MS/MS spectral library search were used to identify the components (Supplementary Table S2).

### 4.2. Animals and Experimental Design

C57BL/6J male mice of specific pathogen-free grade (16 to 24 weeks old, 24 to 32 g) were obtained from Daehan-Bio link (Kyung-gi do, South Korea). The mice were fed a standard chow diet and water ad libitum, and housed in a thermoneutral environment of 24 ± 2 °C with a 12 h light/12 h dark cycle. After 7 days of acclimation, the mice were randomly divided into six groups (*n* = 10 to 12 per each group): (1) the control group (DW with 0.9% saline injection, *n* = 10), (2) LPS group (DW with LPS injection, *n* = 12), YJT groups ((3) 100, (4) 200, or (5) 400 mg/kg of YJT with LPS injection, *n* = 12 for each group), and (6) glutathione (GSH) group (100 mg/kg of GSH with LPS injection, *n* = 12), respectively. Drugs were dissolved in DW, and LPS was prepared in 0.9% saline and stored at −80 °C. Either DW, YJT, or GSH were orally administrated for the first four days. To induce neuroinflammation, we intraperitoneally (i.p) injected LPS repeatedly with slight modification from our previous study [25]. Briefly, from the 5th to 7th day, we injected LPS (1 mg/kg, i.p. on the 5th to 6th days and 2 mg/kg, i.p. on the 7th day) 4 h after drug administration. The mice were sacrificed under anesthesia on the final day of the experiment after receiving 250 mg/kg Avertin (i.p.). Brain tissues were removed from the mice, and from some brain tissues (*n* = 6 to 9 to each group), hippocampal tissues were further dissected and stored at −80 °C for further biochemical analysis. The other tissues (*n* = 3 to 4 to each group) were fixed in 10% neutral formalin solution for histopathological analysis. The Daejeon University Institutional Animal Use and Care Committee approved the entire animal experiment procedure (Approval ID: DJUARB2020-032, Daejeon, Korea). The animal experiment was carried out in compliance with the Guide for the Care and Use of Laboratory Animals published by the United States National Institutes of Health (NIH).

### 4.3. Preparation of Brain Tissue Samples and Dissection of the Hippocampus

All mice were euthanized 18 h after the last LPS injection. We drew whole blood from the abdominal vein and collected sera by centrifugation at 3000× rpm at 4 °C for 15 min. Hippocampal tissue was further dissected from the fresh whole brain and stored in a −80 °C freezer or a liquid nitrogen gas tank for other experiments (*n* = 6 to 9 for whole brain tissue samples to each group). After cardiac perfusion, some of the whole brain tissues were fixed in 10% neutral formalin solution for immunohistochemistry (IHC) analysis. For hippocampal samples (*n* = 6 to 9 to each group), we homogenized them with radio-immunoprecipitation assay (RIPA) buffer (Thermo Fisher SCIENTIFIC, Cat. No; 89900, Waltham, MA, USA) or TRIZOL reagent (Sigma-Aldrich, T-9424-200ML, St. Louis, MO, USA) to acquire protein lysates or messenger ribonucleic acid (mRNA) samples, respectively.

### 4.4. Assessments of Biochemical Assays

We obtained hippocampal homogenates from stored hippocampal tissue (*n* = 6 to 9 in each group) to verify parameters for oxidative stress and analyze antioxidant components. We used commercial kits for all assays, and all measurements were taken according to the manufacturer’s instructions. The degree of lipid peroxidation was measured by the level of malondialdehyde (MDA) using Lipid Peroxidation Colorimetric/Fluorometric Assay Kit (Catalog ^#^ K739, BioVision Inc., Milpitas, CA, USA). The assay’s final products were analyzed and quantified using a spectrophotometer (SoftMax, Ver. 5.4, Molecular Devices, Sunnyvale, CA, USA) at a wavelength of 530 nm. Total GSH content was determined using GSH Assay Kit (Catalog ^#^ CS0260, Sigma-Millipore) and the absorbance was calculated at a wavelength of 405 nm using the spectrophotometer (VersaMax. Ver. 5.1, SoftMax). Superoxide dismutase (SOD) activities were measured using a commercially available kit. As standards, dilutions of bovine erythrocyte Sod (Sigma-Aldrich, St. Louis, MO, USA) in concentrations ranging from 0.01 to 50 U/mL were used. Amplex™ Red Catalase Assay Kit (Catalog ^#^ A22180, Thermo Fishers, Waltham, MA, USA) was used to measure catalase activities. The absorbance was measured at a wavelength of 450 nm using a plate reader.

### 4.5. IHC Analysis

IHC was used to determine hippocampal neuroinflammation markers by detecting microglial cell markers such Myeloperoxidase (MPO) and CD11b according to the previous study [21]. We used 10% formalin to fix brain tissues, and paraffin to embed them. The embedded sections were sliced into 4-µm-thick slices. The slides were deparaffinized in xylene, rehydrated in DW, rinsed in gently running tap water, heated in the microwave for 5 min using sodium citrate buffer (pH 6.0, 10 mM), then cooled. After rinsing, the slides were immersed for 10 min in 3% hydrogen peroxide before being rinsed again. The slides were then kept overnight at 4 °C with primary antibodies such as anti-rabbit-myeloperoxidase (MPO)-antibody (1:200; Invitrogen, Waltham, MA, USA) and anti-CD11b Recombinant Rabbit Monoclonal Antibody (RM290) (Catalog ^#^ MA5-27894 1:200; Invitrogen, Waltham, MA, USA), respectively. The next day, the primary antibodies were washed out and the slides were incubated with biotinylated secondary antibody, then with avidin-biotin-peroxidase complex using commercially available kit (VECTASTAIN^®^ Elite^®^ ABC Universal Kit, Peroxidase, R.T.U (Horse Anti-Mouse/Rabbit IgG, ^#^ PK-7200, VETOR LABORATORIES, CA, USA). The immunoreactive signal was visualized using a 3,3′-diaminobenzidine (DAB) substrate (ImmPACT^®^ DAB Substrate Kit, Peroxidase, ^#^ SK-4105 VETOR LABORATORIES). An optical microscope (Leica Microsystems, Wetzlar, Germany) with magnifications of 40× and 100× was used to examine the slides. The intensity of positive signals was quantified using the Image J 1.64 software under the 100× magnification condition (NIH, Bethesda, MD, USA).

### 4.6. Western Blot Analysis

For Western blot analysis, hippocampus samples were homogenized using RIPA buffer, which contains proteinase inhibitors (cOmplete™ Protease Inhibitor Cocktail, REF 05056489001, Roche, Indianapolis, IN, USA). Briefly, protein samples (50 µg per each sample) were loaded onto the gel for sodium dodecyl sulfate polyacrylamide gel electrophoresis (SDS-PAGE), and then transferred to polyvinylidene fluoride (PVDF) membranes (Millipore, Billerica, MA, USA). For nonspecific binding, the membranes were incubated for 1 h in PBS containing 5% nonfat powdered milk and 0.1% Tween 20. After nonspecific binding was blocked, the membranes were incubated with the primary antibodies: anti-mouse-MDA (Invitrogen, ^#^ MA5-27559, 1:1000), anti-mouse-4-hydroxypeoneal (R&D, ^#^ MAB-1676, 1:1000), anti-mouse-nitrotryptophan (Jaica, ^#^ MNW-020P, Japan, 1:1000), anti-mouse-sirtuin6 (Sirt6) antibody (Invitrogen, ^#^ MA1-214, 1:1000), anti-rabbit-inducible nitric oxide synthase (iNOS) antibody (Invitrogen, ^#^ PA3-030A), anti-rabbit-nuclear factor kappa-light-chain-enhancer of activated B cells (Nf-κb) antibody (Abcam, ^#^ ab16502, 1:1000), anti-rabbit-nuclear factor of kappa light polypeptide gene enhancer in B-cells inhibitor, alpha (IκBα) antibody (CST, ^#^ 9242S, 1:1000), anti-rabbit-toll-like receptor 4 (TLR4) antibody (Novus, ^#^ 76B357.1, 1:1000), and anti-mouse-Actinin (SCBT, sc-166524, 1:3000), anti-rabbit-NRF2/Nfe2l2 antibody (Proteintech, ^#^ 6396-1-AP, 1:1000), anti-mouse- HMOX1 antibody (HO-1) (Invitrogen, ^#^ MA1-112, 1:1000), anti-rabbit-Histone H3 (D1H2) antibody (CST, ^#^ 4499, 1:1000), and anti-rabbit-Acetyl-Histone H3 (Lys56) antibody. After incubating all the antibodies listed above in blocking solution overnight at 4 °C, the blots were rinsed and incubated at RT with a horseradish peroxidase (HRP)-conjugated secondary antibody for 1 h. A Thermo Fisher Scientific Image Reader and a chemiluminescent detection reagent (Thermo Fisher Scientific) were used to measure peroxidase activity [20,21]. For all assays, we used α-actinin as a reference protein. The protein intensity for each blot was measured by Image J 1.64 software as normalized in inner control protein.

### 4.7. Gene Expression Analysis

We used mRNA samples (1 μg) isolated from the hippocampus of mice (*n* = 4 to 6 for each group) using Trizol reagent to synthesize complementary deoxyribonucleic acid (cDNA) in a reaction volume of 20 μL, measured with a High-Capacity cDNA Reverse Transcription Kit (Applied Biosystems, Foster City, CA, USA). We used SYBR Green Master Mix (Thermo Fisher Scientific) in an Eppendorf Realplex polymerase chain reaction (PCR) system (Eppendorf North America Inc., Hauppauge, NY, USA) for real-time PCR analysis. Briefly, reactions were carried out with a mixture of total 10 μL, consisting of 5 μL of SYBR Green Supermix, 1 μL of 10 pM primer pairs, 2 μL of RNase free water, and 2 μL of cDNA samples. Each PCR was handled under the manufacturer’s instructions as follows: 2 min at 50 °C for uracil-DNA glycosylase (UDG) activation, 2 min at 95 °C for DNA polymerase, followed by 40 cycles of amplification steps (15 s at 95 °C, 15 s at 60 °C, and 15 s at 72 °C). Gene expression levels were analyzed with the expression level of β-actin as an internal control. The mRNA levels were evaluated and measured with 2^−^^ΔΔ^CT values. Supplementary Table S3 summarizes the primer sequences, product sizes, and annealing temperatures.

### 4.8. Statistical Analysis

We represented all data as mean ± standard error of the mean (SEM). The statistical significance of the differences among the groups was determined using one-way or two-way analysis of variance (ANOVA), with Bonferroni *t*-test as post hoc multiple comparisons using Prism ver. 9.2 (GraphPad, CA, USA). *p* < 0.05 was used to determine statistically significant differences.

## 5. Conclusions

**Figure 8:**
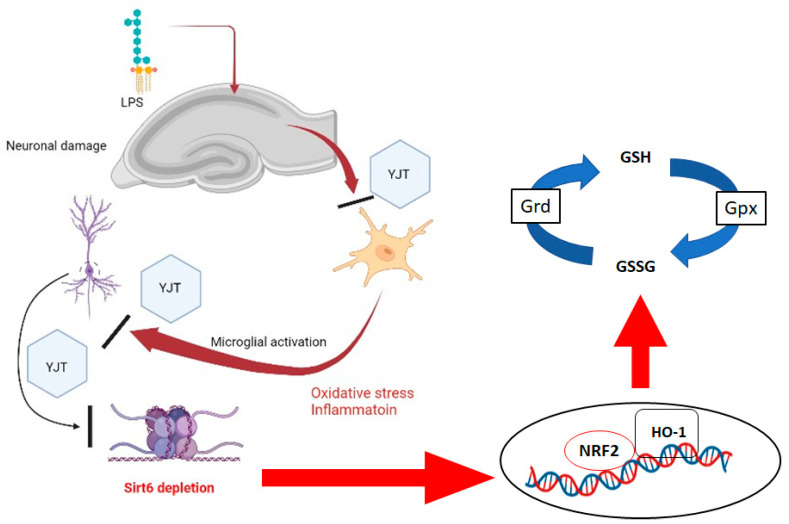
Summary of pharmacological effects of YJT and its underlying mechanism on LPS–induced neuroinflammation using mice model.

In conclusion, the findings in this study show that traditional herbal medicine YJT reduces LPS-induced neurotoxicity via anti-inflammatory activities by suppressing expression levels of Mpo and Emr1 as well as via antioxidant activities by controlling protein levels of MDA, 4-HNE and nitrotryptophan, and via repressing neuroinflammatory signaling pathways by controlling NLRP3, iNOS, p65, Iκbα, and Tlr4. In terms of pharmacological properties, YJT was found to be associated with GSH redox cycle normalization and Sirt6 modulations, which can prevent histone H3 lysine 56 acetylation. Additionally, Nrf2/HO-l signaling pathway was also dependently regulated by Sirt6 levels, which is the main target protein of YJT under the status of neuroinflammation (Figure 8). As a popular traditional Korean medicine, YJT has long been recognized for its anti-aging properties, as the herbal formula is widely used to treat age-related disorders. It is worth noting that YJT in this study had an effect via Sirt6, which is a well-known anti-aging factor. YJT administration could prevent LPS-induced hippocampal injury in mice by preventing Sirt6 down-regulation and restoring its activity in the hippocampus. According to the findings, YJT could be a promising therapeutic agent for age-related neurodegenerative diseases. Further research would be required to identify the corresponding active compounds of the formula.

## Data Availability

Data is contained within the article and Supplementary Materials.

## Supplementary Materials

The following supporting information can be downloaded at: https://www.mdpi.com/article/10.3390/ph15030293/s1; Table S1: Components of YJT and its ratio; Table S2: Quantification analysis of GYJ; Table S3: Primer list for qPCR.
