# Supplementary material for: Pharmacological Effects of Gami-Yukmijihwang-Tang on the Lipopolysaccharide-Induced Hippocampus Oxidation and Inflammation via Regulation of Sirt6
Source: Pharmaceuticals (Basel). 2022 Feb 28;15(3):293. doi: 10.3390/ph15030293 (PMC8955486; doi:10.3390/ph15030293)
Supplement: Supplementary file 1 [file pharmaceuticals-15-00293-s001.zip › pharmaceuticals-1603522-supplementary.pdf]

Supplementary Materials

Table S1. Components of YJT and its ratio.

Herbal Name	Specific Name	Origin	Part of Use	Amount (g)
Rehmanniae Radix Preparata	*Rehmannia glutinosa* Liboschitz var. *purpurea* Makino	Eumseong, South Korea; Processed by Omniherb, Yeongcheon, South Korea	Rhizoma	10
Dioscoreae Rhizoma	*Dioscorea japonica* Thunb.	Andong, South Korea	Rhizoma	10
Corni Fructus	*Cornus officinalis* Sieb. et Zucc.	Gurye, South Korea	Fructus	10
Poria cocos Sclerotium	*Poria cocos* Wolf	Bonghwa, South Korea	Mycelium	10
Gastrodiae Rhizoma	*Gastrodia elata* Blume	Muju, South Korea	Rhizoma	10
Mori Fructus	*Morus alba* Linné	China; Purchased from Omniherb, Yeongcheon, South Korea	Fructus	10
Lycii Fructus	*Lycium chinense* Miller	Cheongyang, South Korea	Fructus	10
Schisandrae Fructus	*Schisandra chinensis* (Turcz.) Baillon	Mungyeong, South Korea	Fructus	10
Cervi Parvum Cornu	*Cervus elaphus sibiricus*	Russia; Purchased from Daehan Bio Pharm Co., Ltd., Hwaseong, South Korea	Cervus	5
Hominis Placenta	*Homo sapiens L.*	South Korea; Manufactured by Kyung Nam Pharm Co., Ltd., Uiryeong, South Korea	Placenta	1
Total				86

Table S2. Quantification analysis of GYJ

RT (min)	m/z ([M + H]$^+$)	Formulae	ppm	ID	Source
1.07	104.1072	C_5H1_3ON	1.723	Choline cation	*Wolfiporia extensa*
1.22(-)	135.0291	$C_4H_6O_5$	2.223	Malic acid	*Lycium chinese Miller*
1.48	268.1047	$C_{10}H_{13}O_4 N_5$	2.684	2'-Deoxyguanosine	*Wolfiporia extensa*
1.48	136.0621	$C_5H_6 N_5$	2.192	adenine	*Wolfiporia extensa*
1.49(-)	193.0347	$C_6 H_8O_7$	2.440	Citric acid	*Lycium chinese Miller*
1.64	132.1022	$C_6 H_{14}O_2N$	2.383	Leucine	*Cervus elaphus sibericus*
2.21	166.0866	$C_9 H_{11}O_2N$	2.197	Phenylalanine	*Cervus elaphus sibericus*
2.29	127.0393	$C_6H_7O_3$	2.514	5-hydroxymethyl-2-furfural (5-HMF)	*Rehmannia glutinosa Liboschitz ex Steudel*
4.37	355.1034	$C_{16}H_{19}O_9$	2.820	Chlorogenic acid	*Lycium chinese Miller* *Morus alba Linné*
4.55	407.1564	$C_{17}H_{27}O_{11}$	4.033	Morroniside	*Cornus officinalis Siebold & Zucc.*
5.68	391.1608	$C_{17}H_{27}O_{10}$	2.292	loganin	*Cornus officinalis Siebold & Zucc.*
6.70	611.1622	$C_{27}H_{31}O_{16}$	2.518	Rutin	*Lycium chinese Miller* *Morus alba Linné*
7.55	625.1782	$C_{28} H_{33}O_{16}$	3.005	Narcissin	*Lycium chinese Miller*
8.14	543.1724	$C_{24}H_{31}O_{14}$	2.868	Cornuside	*Cornus officinalis Siebold & Zucc.*
9.17	447.0932	$C_{21} H_{19}O_{11}$	2.219	Baicalin	*Scutellaria baicalensis*

10.39	595.2035	$C_{28}H_{35}O_{14}$	2.349	Poncirin	*Poncirus trifoliata Rafinesque*
13.44	823.4135	$C_{42}H_{63}O_{16}$	2.912	glycyrrhizin	*Dioscorea batatas Decaisne*
15.30	433.2232	$C_{24}H_{33}O_{7}$	2.563	Schisandrol A	*Schisandra chinensis (Turcz) Baill.*
17.15	515.2286	$C_{28}H_{35}O_{9}$	2.059	Gomisin B (Schisantherin B)	*Schisandra chinensis (Turcz) Baill.*
17.24	501.2495	$C_{28}H_{37}O_{8}$	2.385	Angeloylgomisin H	*Schisandra chinensis (Turcz) Baill.*
17.80	401.1966	$C_{23}H_{29}O_{6}$	1.807	Schisandrin B	*Schisandra chinensis (Turcz) Baill.*
20.92	496.3408	$C_{24}H_{51}O_{7}$ NP	2.003	LysoPC 16:0	*Cervus elaphus sibericus*
21.40	417.2280	$C_{24}H_{33}O_{6}$	2.025	Schisandrin A	*Schisandra chinensis (Turcz) Baill.*
23.13	524.3717	$C_{26}H_{55}O_{7}NP$	1.209	LysoPC 18:0	*Cervus elaphus sibericus*

Table S3. Primer list for qPCR

Gene	Species	Primer Sequence (Forward and Reverse)	Application
Catalase	Mouse	5′- TCA CCC ACG ATA TCA CCA GA -3′<br>5′- AGC TGA GCC TGA CTC TCC AG -3′	qPCR
Sod2	Mouse	5′- GGT GGC GTT GAG ATT GTT CA -3′<br>5′- CCC AGA CCT GCC TTA CGA CTAT -3	qPCR
Mpo	Mouse	5′- GCC AAG GCC TTT CAA TGT TA-3′<br>5′- TCA CGT CCT GAT AGG CAC AG-3′	qPCR
Ccl2<br>*(F4/80)*	Mouse	5′- CCC AAT GAG TAG GCT GGA GA-3′<br>5′- TCT GGA CCC ATT CCT TCT TG-3′	qPCR
Tnfa	Mouse	5′- GGC CTC CCT CTC ATC AGT TC-3′<br>5′- CAC TTG GTG GTT TGC TAC GA-3′	qPCR
Il1b	Mouse	5′- TGT GAA ATG CCA CCT TTT GA-3′<br>5′-GGT CAA AGG TTT GGA AGC AG-3′	qPCR
Il6	Mouse	5′-CAA AGC CAG AGT CCT TCA GAG-3′<br>5′-GAG CAT TGG AAA TTG GGG TA-3′	qPCR
Gpx3	Mouse	5′- GCT TGG TCA TTC TGG GCT TC-3′<br>5′- CCC ACC TGG TCG AAC ATA CT-3′	qPCR
Gsr	Mouse	5′- AGC CGC CTG AAC ACC ATC TA -3′<br>5′-GAT GTG TGG AGC GGT AAA CTT TT -3′	qPCR
Gssh	Mouse	5′-TGT GCC CTT TTA CCC TCT TCC T -3′<br>5′-TCT TTG GAG TGT GGG AAT GGA-3′	qPCR
Sirt6	Mouse	5′-ACG TCA GAG ACA CGG TTG TG-3′<br>5′-CCT CTA CAG GCC CGA AGT C-3′	qPCR
β-actin	Mouse	5′- GGC ACCACACCTTCTACAATGA-3′<br>5′- ATCTTTTCACGGTTGGCCTTAG-3′	qPCR
